# Stroboscopic lighting with intensity synchronized to rotation velocity alleviates motion sickness gastrointestinal symptoms and motor disorders in rats

**DOI:** 10.3389/fnint.2022.941947

**Published:** 2022-07-28

**Authors:** Yuqi Mao, Leilei Pan, Wenping Li, Shuifeng Xiao, Ruirui Qi, Long Zhao, Junqin Wang, Yiling Cai

**Affiliations:** Department of Nautical Injury Prevention, Faculty of Navy Medicine, Naval Medical University, Shanghai, China

**Keywords:** stroboscopic light, motion sickness, gastrointestinal symptoms, motor disorders, Fos protein

## Abstract

Motion sickness (MS) is caused by mismatch between conflicted motion perception produced by motion challenges and expected “internal model” of integrated motion sensory pattern formed under normal condition in the brain. Stroboscopic light could reduce MS nausea symptom via increasing fixation ability for gaze stabilization to reduce visuo-vestibular confliction triggered by distorted vision during locomotion. This study tried to clarify whether MS induced by passive motion could be alleviated by stroboscopic light with emitting rate and intensity synchronized to acceleration–deceleration phase of motion. We observed synchronized and unsynchronized stroboscopic light (SSL: 6 cycle/min; uSSL: 2, 4, and 8 cycle/min) on MS-related gastrointestinal symptoms (conditioned gaping and defecation responses), motor disorders (hypoactivity and balance disturbance), and central Fos protein expression in rats receiving Ferris wheel-like rotation (6 cycle/min). The effects of color temperature and peak light intensity were also examined. We found that SSL (6 cycle/min) significantly reduced rotation-induced conditioned gaping and defecation responses and alleviated rotation-induced decline in spontaneous locomotion activity and disruption in balance beam performance. The efficacy of SSL against MS behavioral responses was affected by peak light intensity but not color temperature. The uSSL (4 and 8 cycle/min) only released defecation but less efficiently than SSL, while uSSL (2 cycle/min) showed no beneficial effect in MS animals. SSL but not uSSL inhibited Fos protein expression in the caudal vestibular nucleus, the nucleus of solitary tract, the parabrachial nucleus, the central nucleus of amygdala, and the paraventricular nucleus of hypothalamus, while uSSL (4 and 8 cycle/min) only decreased Fos expression in the paraventricular nucleus of hypothalamus. These results suggested that stroboscopic light synchronized to motion pattern might alleviate MS gastrointestinal symptoms and motor disorders and inhibit vestibular-autonomic pathways. Our study supports the utilization of motion-synchronous stroboscopic light as a potential countermeasure against MS under abnormal motion condition in future.

## Introduction

Motion sickness (MS) is a common disorder caused by real motion during traveling (e.g., in boats, cars, and airplanes) or by illusory motion during virtual reality immersion (e.g., watching 3D video films) (Lackner, 2014; Nalivaiko et al., 2014; Zhang et al., 2016). The main symptoms of MS include autonomic reactions (i.e., nausea, vomiting, pallor, cold sweating, and hypersalivation), motor disorders (hypoactivity and balance disturbance), and sopite syndrome (i.e., drowsiness, lethargy, and persistent fatigue) (Lackner, 2014). Mismatch between sensory information at present (conflicted visual, vestibular, and/or proprioceptive signals) and the anticipated “internal model” (integrated motion sensory pattern formed under normal condition) leads to MS responses (Reason, 1978; Golding, 2006; Bertolini and Straumann, 2016; Zhang et al., 2016). For example, stationary subjects who view the interior of an optokinetic drum often experience visual MS symptoms, as visual inputs indicating self-motion are inconsistent with vestibular input indicating no self-motion (Bubka et al., 2006). Virtual reality vision which produces an illusory sensation of motion could elicit cybersickness when proprioceptive and vestibular organs provide no cues of self-motion (Gallagher and Ferre, 2018). MS often develops in people sitting on the back seat in a moving vehicle or being swung in an enclosed cabin on a ship without external visual references, while providing a clear external view (like viewing the horizon at sea) seems to be an effective measure to re-balance visual and vestibular afference and alleviate MS symptoms during unexpected motion stimulation (Griffin and Newman, 2004; Brainard and Gresham, 2014). These observations suggested that simultaneous visual and vestibular input with contradictory or uncorrelated pattern could induce MS (Schmal, 2013).

Combined visual and vestibular cues in synchronization could provide greater estimate of head direction and velocity than either cue available alone under both linear and angular motion conditions (Fetsch et al., 2010; Keshavarzi et al., 2022). Optokinetic nystagmus rotation that had a phase lead relative to chair rotation could elicit MS symptoms in both motion-susceptible and non-susceptible subjects (Dai et al., 2011), while oculo-vestibular re-coupling using galvanic vestibular stimulation to produce head motion perceptions that are synchronized to the speed and direction of a moving visual field can mitigate MS in subjects exposed to flight simulation (Cevette et al., 2012). It suggested that re-balance of visual and vestibular afference to promote rapid visuo-vestibular sensory integration can reduce conflict that causes MS (Carriot et al., 2015; Chen et al., 2017). Based on this theory, stroboscope illumination has been used to prevent MS by reducing the impact of unreliable and disturbed visual cues that conflict with vestibular information. An early study showed that stroboscopic light prevented MS symptoms in subjects wearing distorting optics and simultaneously making free navigation around the laboratory which always induces overt visuo-vestibular conflict and MS (Jones and Mandl, 1981). Stroboscopic illumination also reduced the severity of MS symptoms in subjects who are reading text through reversing prisms while making active pitch head movements (Reschke et al., 2006). Given that visuo-vestibular re-coupling can reduce sensory conflict, we hypothesized that stroboscopic illumination with the lighting intensity synchronized dynamically with motion velocity could be a benefit for re-balancing visuo-vestibular signals and counteracting MS induced by passive motion stimulation.

Physical motion-induced MS in humans can be assessed by rating cardinal signs and symptoms such as gastrointestinal disturbance (epigastric discomfort, nausea, vomiting, and salivation), thermoregulatory disruption (cold sweating), alterations in arousal as well as dizziness, vertigo, and headache (Cha et al., 2021). As the lack of vomiting reflex, varieties of behavioral measurements have been widely used to assess multi-dimensional responses of MS in rodents. Conditioned gaping response that can be elicited during exposure to a context previously paired with nauseant agents or provocative motion can be alleviated by corresponding anti-emetic and anti-MS drugs, making it plausible to serve as a rat model of conditioned nausea (Limebeer et al., 2008; Parker, 2014; Zhou et al., 2017; Qi et al., 2019; Rock et al., 2022). In addition, defecation response and decreased spontaneous locomotion activity were also observed in rats and mice exposed to whole-body rotation (Ossenkopp and Frisken, 1982; Ossenkopp et al., 1994; Yu et al., 2007; Limebeer et al., 2008; Morita et al., 2017; Wang et al., 2017; Deshetty et al., 2021). Balance disorder characterized by the decline in the time taken traversing balance beam was also elicited by passive motion stimulation in both rats and mice (Tung et al., 2014; Zhou et al., 2017; Manno et al., 2020; Zhong et al., 2022). Similar disruption in balance control and locomotion activity has been reported in MS vehicle passengers and in astronauts exposed to altered gravitational environment during space flight or following landing (Le et al., 2020; Carriot et al., 2021). This evidence suggested that rodents could also manifest multiple MS-related signs and symptoms, especially gastrointestinal and motor disorders, similar to those observed in humans.

In this study, we tried to investigate the efficacy of stroboscopic lighting in alleviating MS-related behavior responses in rats exposed to Ferris wheel-like rotation. The effects of stroboscopic light synchronized or unsynchronized to the acceleration–deceleration phase of rotation were examined, and the influence of color temperature and lighting intensity was also observed. MS-related gastrointestinal responses were assessed by measuring conditioned gaping and defecation responses due to the absence of vomiting reaction in rodents (Wang et al., 2012, 2017; Zhou et al., 2017; Qi et al., 2019; Devuono et al., 2020). Motor and balance performance were evaluated using spontaneous locomotion test and balance beam test (Manno et al., 2020). Furthermore, numerous studies have reported that activation of vestibular-autonomic related brain areas was critical for the production of autonomic MS manifestations (Yates et al., 1999; Pompeiano et al., 2004; Balaban et al., 2014; Nalivaiko et al., 2014). Provocative motion stimulation could increase the expression of Fos protein (a marker of neuronal activity) in vestibulo-autonomic nuclei such as the caudal vestibular nucleus (CVN) including caudal medial and spinal vestibular nucleus, the nucleus of solitary tract (NTS), the parabrachial nucleus (PBN), the central nucleus of amygdala (CeA), and in stress-related areas such as the locus coeruleus (LC) and the paraventricular nucleus of hypothalamus (PVN) (Nakagawa et al., 2003; Cai et al., 2010; Balaban et al., 2014; Morita et al., 2017). These structures constitute neural networks that were believed to contribute to MS induced by vestibular stimulation (Balaban et al., 2014; Zhou et al., 2017). This study also observed alteration of Fos expression pattern in these regions to clarify vestibular neural pathways that were potentially regulated by stroboscopic lighting.

## Materials and methods

### Animals and ethnics

Adult male Sprague-Dawley rats (weight: 230–250 g) were purchased from Shanghai Sippr-BK laboratory animal Co. Ltd. They were individually housed (temperature: 22 ± 2°C and lighting: 8:00–20:00) with free access to food and distilled water. Before the experiment, all animals were adapted to the lab environment for at least 2 weeks. All animal protocols and procedures complied with the Guide for the Care and Use of Laboratory Animals (National Research Council, 2011) and were approved by the Ethics Committee for Animal Experimentation of the Naval Medical University (Shanghai, China).

### Rotation device and procedures

**Figure 1:**
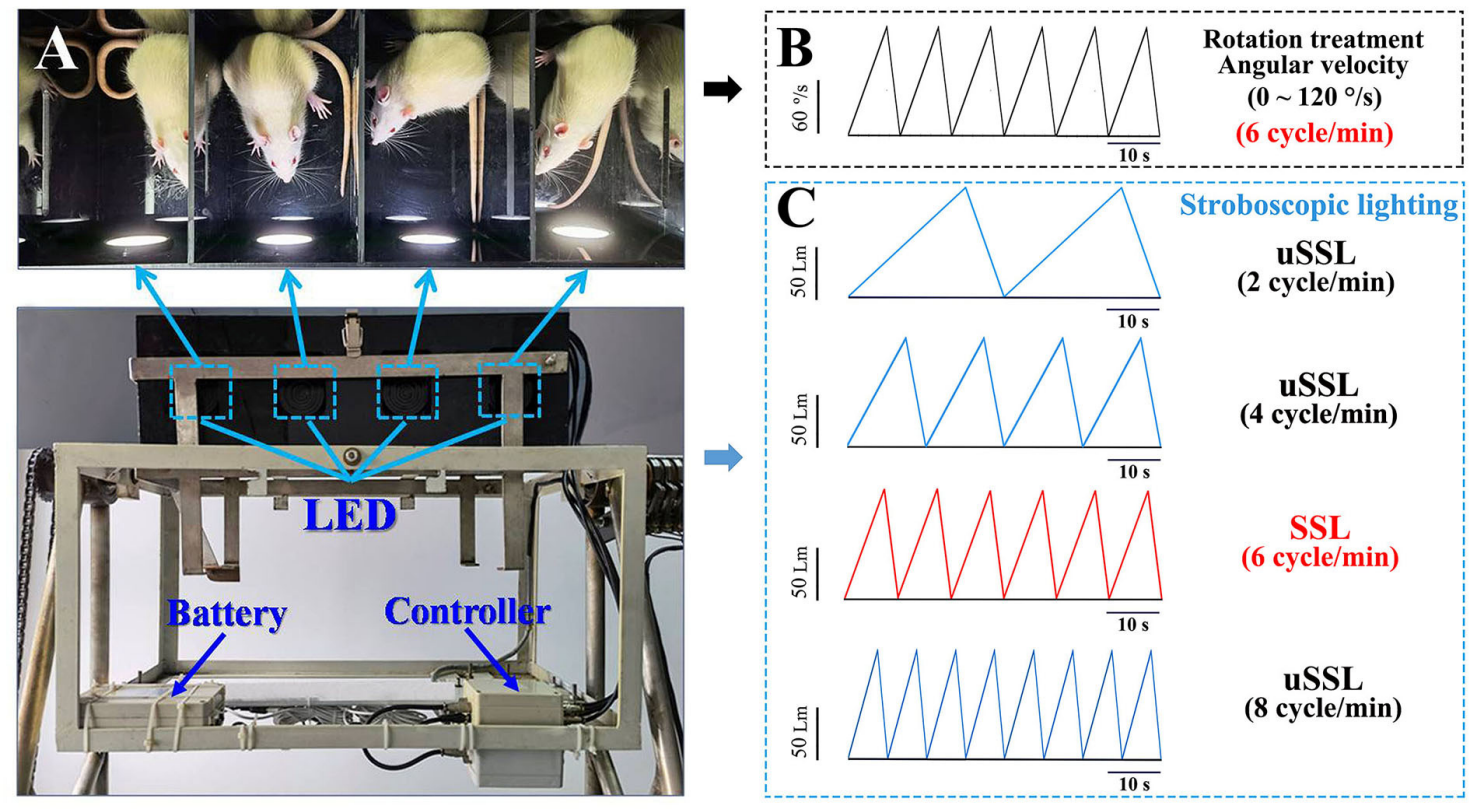
Images illustrating the manner of stroboscopic lighting treatment during the Ferris wheel-like rotation in rats. **(A)** Top view of the plexiglass box of the rotation device that was equipped with four sets of LEDs mounted on the side wall of four separate chambers. The LEDs were connected to a controller which was used to set lighting parameters prior to the experiment. The stroboscopic light system was powered by a lithium battery fixed on the frame of the rotation device. **(B)** The schematic diagram showing the change of rotation angular velocity (120°/s at peak) and **(C)** stroboscopic light intensity (100 Lm at peak) as a function of time during rotation treatment in Experiment 1. LED emitting rate was set in synchronized (6 cycle/min, light intensity was represented by red lines) or unsynchronized (2, 4, and 8 cycle/min; light intensity was represented by blue lines) manners relative to the acceleration–deceleration rotation circle. The color temperature was set at 6,000 and 3,000 K as cool and warm light, respectively. The peak light intensity was adjusted as requested in Experiment 2 (50, 100, or 200 Lm) and Experiment 3 (100 Lm). LED, light-emitting diode; SSL, synchronized stroboscopic light; uSSL, unsynchronized stroboscopic light.

The Ferris wheel-like rotation device was modified based on the one created by Crampton and Lucot (1985) and has been used to establish MS rat model in our previous studies (Wang et al., 2012, 2017; Zhang et al., 2016; Zhou et al., 2017). It is composed of two plexiglass boxes suspended on two metal frames. Each box was separated into four chambers (length × width × height: 26 × 22.5 × 20 cm for each chamber) to contain animals individually without restraint to minimize general stress (Figure 1A). During rotation, the plexiglass box accelerated in a clockwise direction at 16°/s^2^ to reach an angular velocity of 120°/s and then began to decelerate at 48°/s^2^ to reach 0°/s. An acceleration–deceleration circle lasted 10 s (6 cycle/min, 0.1 Hz in frequency; Figure 1B). Animals in rotation (Rot) groups received Rot treatment continuously for 2 h, while those in static (Sta) control groups were kept in the plexiglass chambers next to the rotation device only receiving the noise and the vibration produced by the device but not being rotated. Animals in the non-lighting Rot and Sta control group received rotation or static control treatment, respectively, in complete darkness.

### Stroboscopic light system

The stroboscopic light system consists of four sets of light-emitting diode (LED) mounted on the center of the side wall of the light-reflective plexiglass chamber to provide efficient stroboscopic illumination in the whole chamber during the experiment (Figure 1A). Each LED was connected to a controller (Shanghai Lansheng Electronic Technology Co., Ltd) that was used to set stroboscopic parameters (light intensity, color temperature, and emitting rate) with a built-in software. Light intensity is quantified by luminous flux with the unit of measure–lumen (Lm), while color temperature is expressed by the unit of measure–kelvins (K), with “cool color” defined as >5,000 K and “warm color” as 2,700–3,000 K (Archer, 2018). In this study, environment illumination was set at about 150 lux in the room where the animals were raised according to the recommendation (130–325 lux) of the Guide for the Care and Use of Laboratory Animals (National Research Council, 2011). To avoid irritation effect, stroboscopic lighting intensity was set at 50, 100, or 200 Lm which produced illumination at about 37.5, 75, and 150 lux in maximum at the middle of the rotation chamber during the experiment. For each lighting cycle, light intensity increased linearly as a function of time to reach the peak (Experiments 1 and 3: 100 Lm; Experiment 2: 50, 100, or 200 Lm) and then started to decrease to get to 0 Lm. Emitting rate was set at 6 cycle/min (0.1 Hz) for synchronized stroboscopic light (SSL) with rate and intensity dynamically synchronous with angular velocity of rotation, or at 2 cycle/min (0.033 Hz), 4 cycle/min (0.067 Hz), or 8 cycle/min (0.133 Hz) for unsynchronized stroboscopic light (uSSL) serving as lighting control conditions (Figure 1C). Color temperature was set at 6,000 and 3,000 K as cool and warm light (CL and WL), respectively.

### Experimental design and grouping

#### Experiment 1

A total of 300 rats were used and evenly assigned to three batches of tests which were carried out to examine gastrointestinal symptoms (conditioned gaping and defecation responses), spontaneous locomotion activity, and balance beam performance. For all three tests, animals were randomly divided into eight Rot groups including four CL and four WL groups (one SSL and three uSSL groups for each color temperature setting), as well as one non-lighting group and one Sta control group (*n* = 10 for each group in each test).

#### Experiment 2

Experiment 1 showed that SSL (6 cycle/min) was effective for relieving Rot-induced gastrointestinal responses (defecation and conditioned gaping) as well as hypoactivity and balance disturbance, regardless of color temperature. In contrast, uSSL (4 and 8 cycle/min) was only effective for alleviating defecation. In this part, we observed the peak intensity-dependent effects of SSL on MS responses. One hundred and eighty rats were used for assessing gastrointestinal symptoms, spontaneous locomotion activity, and balance beam performance. For each test (*n* = 60), animals were randomly divided into five groups: three SSL (6 cycle/min) Rot groups (WL; 50, 100, and 200 Lm), a non-lighting group, and a Sta control group (*n* = 12 in each group for each test).

#### Experiment 3

In this part, we tried to identify target vestibulo-autonomic neural pathways potentially regulated by SSL during MS. Eighteen animals were randomly divided into the following groups: four Rot groups treated with SSL or uSSL (WL; 100 Lm), as well as a Rot non-lighting group and a Sta control group (*n* = 3 in each group). Animals were killed immediately after Rot or Sta treatment for further immunohistochemistry analysis.

### Behavioral test

#### Gastrointestinal symptoms

For conditioned gaping test, 10 ml plastic tubes filled with cotton strips impregnated with vanilla extract were attached to the inner side wall of the plexiglass box to continuously release the vanilla smell. During conditioning stage, animals received three sessions of Rot or Sta treatment (unconditioned) paired with odor stimulation (conditioned) with 24-h interval. During the testing stage, animals were only exposed to the odor for 2 h and the gaping behaviors were recorded simultaneously by digital cameras (SONY, HDR-PJ670, Japan) equipped below chambers with transparent plexiglass bottom floors. A conditioned gaping behavior is defined as a rapid and extensive opening of the mandible with incisors completely visible (Rock et al., 2019). The defecation response was measured via counting the number of fecal granules deposited by each rat in the plexiglass container immediately after the first session of conditioning trial.

#### Spontaneous locomotion performance

Immediately after Rot or Sta treatment, animals were taken out of the rotation containers and received spontaneous locomotion recording in an enclosed soundproof room following the same procedures as described in our previous study (Zhou et al., 2017; Qi et al., 2019). The apparatus consisted of a dark 40 × 40 × 45 cm rectangular chamber with the floor marked with a 16 × 16 grid. Each rat was gently placed in the center of the chamber and then left undisturbed for 3 min. Locomotion tracking of the animals was recorded by an infrared digital video camera of the behavior data collecting system (RD1112-IFO-R-4, Mobiledatum, Shanghai, China). Total distance traveled and immobile duration were measured with a commercially available analysis software (EthoVision XT 8.5, Noldus, Netherlands).

#### Balance beam test

Motor coordination was assessed by measuring the time taken to traverse an elevated (90 cm) narrow wooden beam (2.5 × 100 cm) and enter a black plastic box (15 × 15 × 8 cm) at the opposite end. Before testing, each animal was trained daily for 5–9 consecutive days to achieve a stable performance with the time to cross the balance beam <2.5 s. Immediately after Rotor Sta control treatment, three trials were performed and the average time to cross the beam for each animal was used in the statistical analysis. The animals were allowed a 60 s rest between each trial to reduce stress and fatigue.

### Immunohistochemistry

Animals were anesthetized with an overdose of sodium pentobarbital (100 mg/kg, i.p.) and perfused transcardially with 100 ml chilled saline, followed by perfusion with 500 ml of 0.1 mol/L phosphate buffer (PB, pH 7.4) containing 4% paraformaldehyde. The brains were removed, post-fixed, and then cut into 20-μm thick sections throughout. One out of every three consecutive sections containing the target regions including the CVN (Bregma −11.6 to −12.3 mm), the NTS (Bregma −12.7 to −14.3 mm), the PBN (Bregma −8.7~-10.0 mm), the CeA (Bregma −1.8~-3.6 mm), the LC (Bregma −9.10 to −10.0 mm), and the PVN (Bregma −0.8 to −2.1 mm) was selected and incubated with a mouse anti-Fos IgG (Abcam, Cambridge, UK;1:1,000) for 24 h at 4°C. After washing in PBS, the sections were incubated with biotinylated horse anti-mouse IgG (Vector Laboratories, Burlingame, CA; 1:200) for 4 h. Fos immunolabeling was visualized using ABC immunoperoxidase method according to the manufacturer's instruction (Vector Laboratories, Burlingame, CA, USA). Non-specific immunostaining was ruled out by processing some control sections without the primary antibody. The number of Fos-labeled (Fos-LI) neurons was counted under a light microscope by a rater unaware of the experimental design, and the photographs were taken with a digital camera.

### Statistical analysis

All statistical analyses were performed with the SPSS v22.0 statistical program. The sample size was estimated based on our pilot data using PASS 15 software with the parameters set at β = 0.2 and α = 0.05 in Experiments 1 and 2. Given the possible irritant effects of high light intensity, σ value was increased by 10 and 20% (relative to 50 Lm Rot group) for 100 Lm and 200 Lm Rot groups, respectively, in sample estimation in Experiment 2. Shapiro–Wilk analysis was performed to verify normal distribution of the data, and homogeneity of variances was determined using Levene's test. One-way ANOVA analysis was carried out to examine group difference using grouping factor as independent variable and measured values as dependent variables. Bonferroni's *post-hoc* analysis was used to analyze the difference between each group when a significant main effect was obtained. All data were presented as the mean± S.E. Statistical significance was set at *P* < 0.05.

## Results

### Stroboscopic light at SSL (6 cycle/min) alleviated MS-related gastrointestinal responses

**Figure 2:**
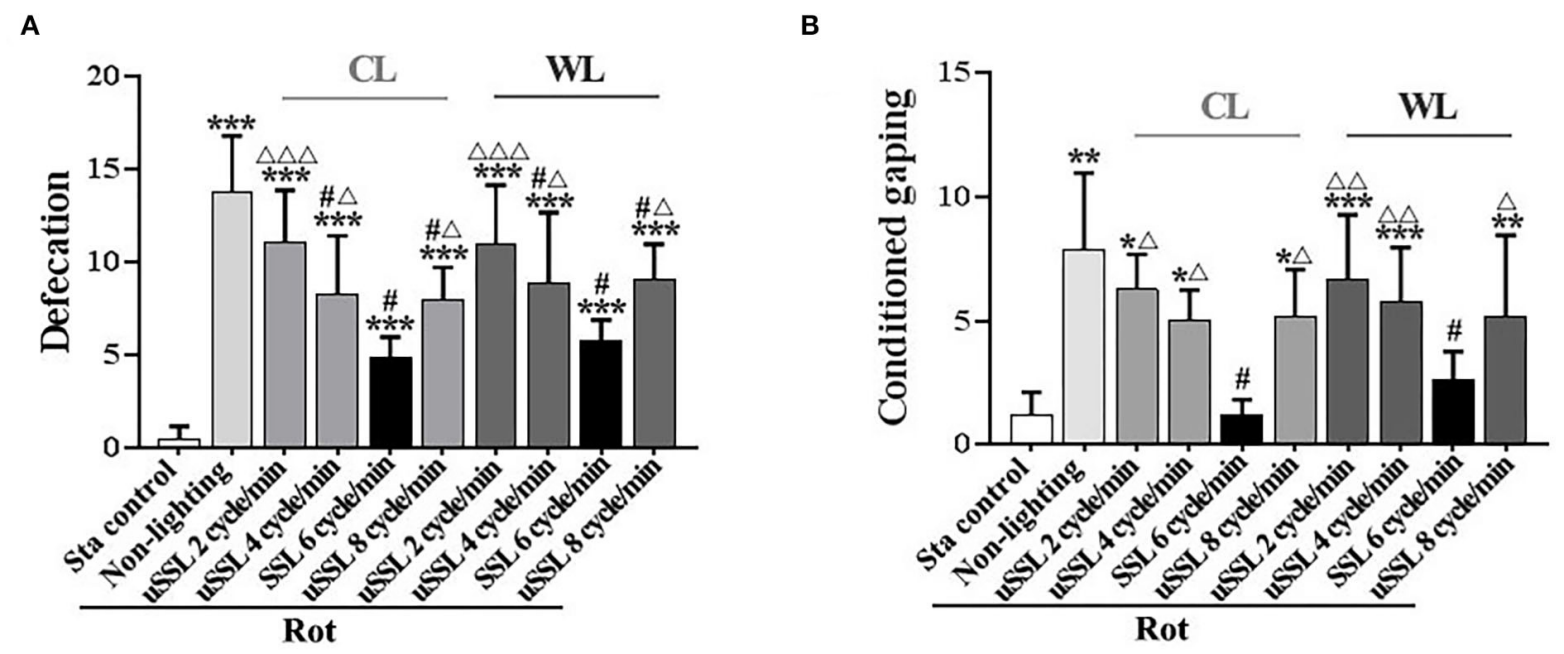
Effects of synchronized (6 cycle/min) or unsynchronized stroboscopic light (2, 4, and 8 cycle/min) at 100 Lm on defecation **(A)** and conditioned gaping **(B)** induced by rotation in rats. Rot, rotation stimulation; Sta, static control treatment; SSL, synchronized stroboscopic light; uSSL, unsynchronized stroboscopic light; CL, cool light; WL, warm light. Data are expressed as mean ± S.E. *P < 0.05, **P < 0.01, ***P < 0.001 vs. the Sta control group; ^#^*P* < 0.001 vs. the Rot non-lighting group; ^Δ^*P* < 0.05, ^ΔΔ^*P* < 0.01, ^ΔΔΔ^*P* < 0.001 vs. the corresponding SSL (6 cycle/min) Rot group with the same color temperature.

Defecation response was induced in all Rot groups compared with the Sta controls [*F*_(9,90)_ = 21.06, *P* < 0.001; *post-hoc*: *P* < 0.001, Figure 2A]. SSL (6 cycle/min) and uSSL (4 and 8 cycle/min) of both CL and WL, but not uSSL (2 cycle/min), partially relieved defecation response in Rot animals compared with the non-lighting controls (*post-hoc*: *P* < 0.001). Animals receiving SSL also showed lower defecation response than those treated with uSSL for both CL and WL (*P* < 0.05 or 0.001). In addition, rotation stimulation led to remarkable conditioned gaping behavior in Rot non-lighting animals [*F*_(9,90)_ = 8.175, *P* < 0.001; *post-hoc*: *P* < 0.01 vs. Sta controls, Figure 2B]. SSL (6 cycle/min) of both CL and WL significantly decreased conditioned gaping responses in Rot animals compared with the non-lighting controls (*P* < 0.05). In contrast, uSSL (2, 4, and 8 cycle/min) groups had higher gaping numbers than Sta controls (*P* < 0.05, 0.01, or 0.001) and the corresponding SSL groups (*P* < 0.05 or 0.01) for both CL and WL. No significant difference was observed between CL and WL groups for each emitting rate in either defecation or conditioned gaping response (Figures 2A,B). There was also no significant difference among uSSL and non-lighting Rot groups for each color temperature.

### SSL (6 cycle/min) alleviated MS-related hypoactivity and balance disturbance

**Figure 3:**
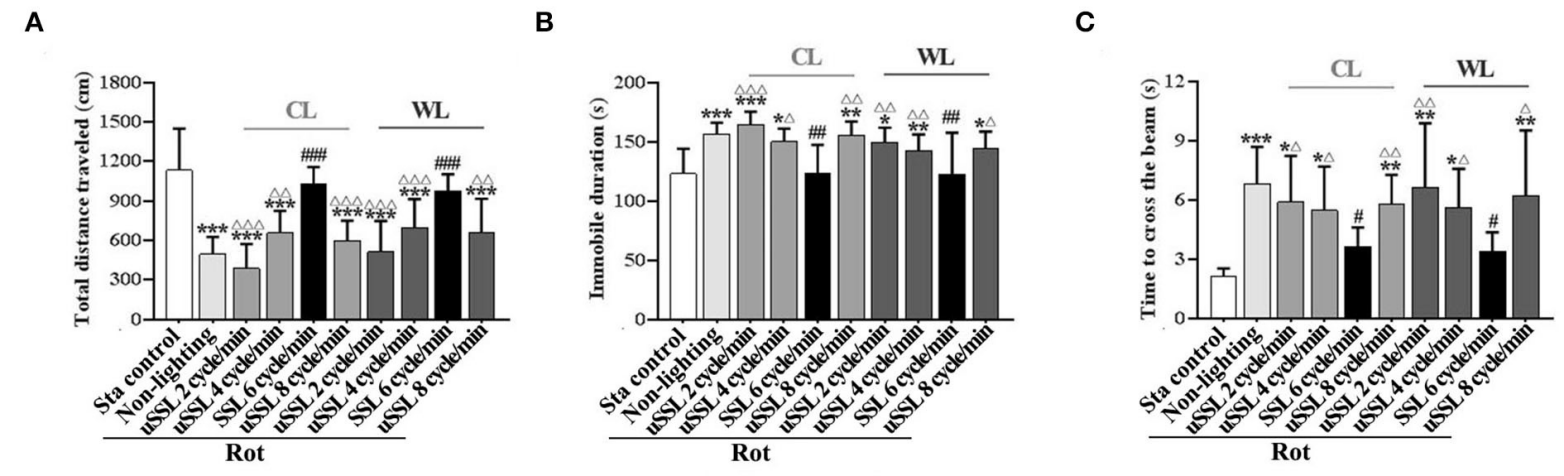
Effects of synchronized (6 cycle/min) or unsynchronized stroboscopic light (2, 4, and 8 cycle/min) at 100 Lm on hypoactivity and balance disturbance induced by rotation in rats. Spontaneous locomotion activity was characterized by total distance traveled **(A)** and immobile duration **(B)**. Motor coordination was assessed by measuring the time to traverse the balance beam **(C)**. Rot, rotation stimulation; Sta, static control treatment; SSL, synchronized stroboscopic light; uSSL, unsynchronized stroboscopic light; CL, cool light; WL, warm light. Data are expressed as mean ± S.E. *P < 0.01, **P < 0.01, ***P < 0.001 vs. the Sta control group; ^#^*P* < 0.05, ^##^*P* < 0.01, ^###^*P* < 0.001 vs. the Rot non-lighting group. ^Δ^*P* < 0.05, ^ΔΔ^*P* < 0.01, ^ΔΔΔ^*P* < 0.001 vs. the corresponding SSL (6 cycle/min) Rot group with the same color temperature.

In spontaneous locomotion test, rotation treatment significantly reduced total distance traveled [one-way ANOVA: *F*_(9,90)_ = 14.27, *P* < 0.001; *post-hoc*: *P* < 0.001; Figure 3A] and increased immobile duration [one-way ANOVA: *F*_(9,90)_ = 7.46, *P* < 0.001; *post-hoc*: *P* < 0.05, 0.01, or 0.001; Figure 3B] in the Rot non-lighting and all uSSL groups compared with the Sta control. Rot-induced hypoactivity was completely relieved by SSL (6 cycle/min) of both CL and WL (total distance traveled: *P* < 0.001; immobile duration: *P* < 0.01), but not alleviated by uSSL (2, 4, or 8 cycle/min) relative to the non-lighting controls. For both color temperatures, SSL groups also showed an increased total distance traveled (*P* < 0.01 or 0.001; Figure 3A) and a decreased immobile duration (*P* < 0.05, 0.01, or 0.001; Figure 3B) compared with the corresponding uSSL groups. There was no difference between CL and WL groups for each emitting rate in either total distance traveled or immobile duration. No significant difference was observed among uSSL group and non-lighting Rot groups for both CL and WL.

In balance performance test, the time to cross the beam was significantly increased in Rot non-lighting group and all uSSL groups compared with the Sta control group [one-way ANOVA: *F*_(9,90)_ = 6.58, *P* < 0.001; *post-hoc*: *P* < 0.05, 0.01, or 0.001; Figure 3C)]. Animals treated with CL and WL at SSL (6 cycle/min) spent less time to traverse the beam than those in the non-lighting group (*P* < 0.05) and the corresponding uSSL groups (*P* < 0.05 or 0.01), while no difference was observed when they were compared with the Sta controls. In contrast, no difference was observed when each uSSL group was compared with the non-lighting Rot group. There was also no difference between CL and WL groups for each emitting rate.

### Intensity-dependent effects of SSL (6 cycle/min) on MS-related gastrointestinal responses

**Figure 4:**
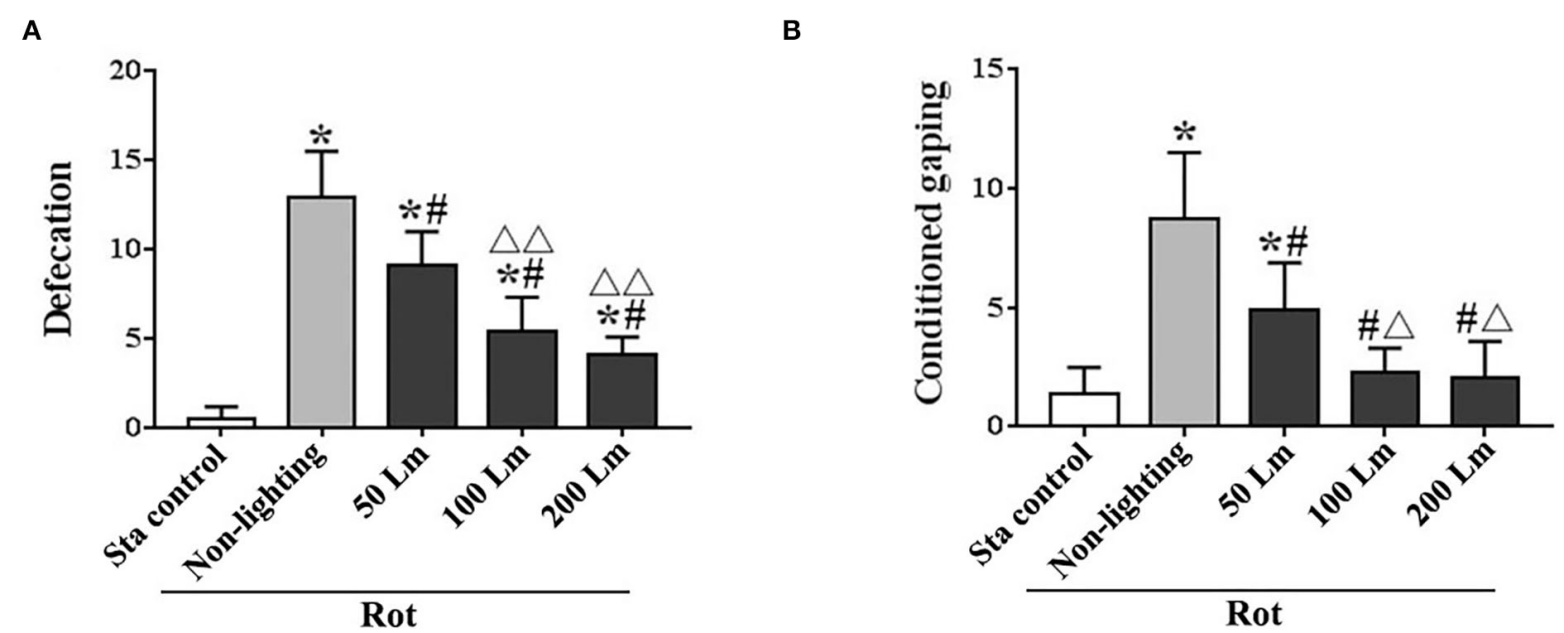
Effects of synchronized stroboscopic light (6 cycle/min, warm light) with different peak intensities (50, 100, and 200 Lm) on defecation **(A)** and conditioned gaping **(B)** induced by rotation in rats. Rot, rotation stimulation; Sta, static control treatment. Data are expressed as mean ± S.E. *P < 0.001 vs. the Sta group; ^#^*P* < 0.001 vs. the Rot non-lighting group. ^Δ^*P* < 0.01, ^ΔΔ^*P* < 0.001 vs. the 50 Lm Rot group.

There were significant differences in the number of fecal granules [*F*_(4,55)_ = 99.99, *P* < 0.001] and the number of conditioned gaping [*F*_(4,55)_ = 34.20, *P* < 0.001] among groups. All Rot groups showed increased defecation responses (*post-hoc*: *P* < 0.001; Figure 4A), while only the Rot non-lighting group and the 50 Lm SSL group showed an increase in conditioned gaping response compared with the Sta control group (*P* < 0.001; Figure 4B). Rot-induced defecation and conditioned gaping were significantly attenuated in SSL groups in an intensity-dependent manner (*P* < 0.001 vs. the non-lighting group). Animals treated with 100 or 200 Lm SSL had lower defecation and conditioned gaping responses than those treated with 50 Lm SSL (*P* < 0.01 or 0.001; Figures 4A,B), while no significant difference was observed between 100 and 200 Lm group.

### Intensity-dependent effects of SSL (6 cycle/min) on MS-related hypoactivity and balance disturbance

**Figure 5:**
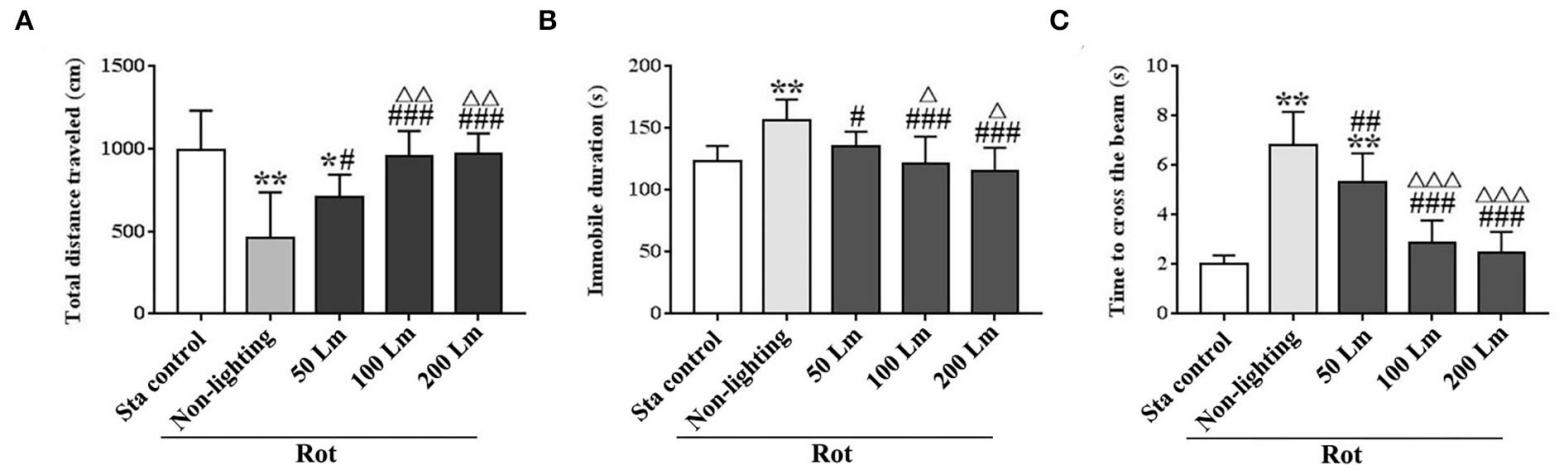
Effects of synchronized stroboscopic light (6 cycle/min, warm light) with different peak intensities (50, 100, and 200 Lm) on hypoactivity and balance disturbance induced by rotation in rats. Spontaneous locomotion activity was characterized by total distance traveled **(A)** and immobile duration **(B)**, while balance performance was characterized by the time to cross the elevated balance beam **(C)**. Rot, rotation stimulation; Sta, static control treatment. Data are expressed as mean ± S.E. *P < 0.05, **P < 0.001 vs. the Sta control group; ^#^*P* < 0.05, ^##^*P* < 0.01, ^###^*P* < 0.001 vs. the Rot non-lighting group; Δ*P* < 0.05, Δ*ΔP* < 0.01, Δ*ΔΔP* < 0.001 vs. the 50 Lm Rot group.

Significant differences in total distance traveled [*F*_(4,55)_ = 16.82, *P* < 0.001] and immobile duration [*F*_(4,55)_ = 11.64, *P* < 0.001] were observed among groups in spontaneous locomotion test (Figures 5A,B). Rot treatment significantly decreased total distance traveled and increased immobile duration in the non-lighting group compared with the Sta control one (*post-hoc*: *P* < 0.001). Rot-induced hypoactivity was significantly attenuated in all three SSL (6 cycle/min) groups (*P* < 0.05 or 0.001 vs. the non-lighting group). It was completely relieved by 100 and 200 Lm SSL (*P* < 0.001 vs. the non-lighting group) for both measurements (Figures 5A,B), but was partially relieved by 50 Lm SSL in total distance traveled (*P* < 0.05 vs. the non-lighting group; *P* < 0.05 vs. the Sta control group; Figure 5A). Rot animals in the 100 and the 200 Lm groups also showed increased total distance traveled (Figure 5A) and decreased immobile duration (Figure 5B) compared with those in the 50 Lm group (*P* < 0.05 or 0.01). There was no difference in either total distance traveled or immobile duration between the 100 and the 200 Lm groups.

In balance performance test, Rot treatment decreased the time to cross the beam in the non-lighting group compared with the Sta control group [*F*_(4,55)_ = 53.96, *P* < 0.001; *post-hoc*: *P* < 0.001; Figure 5C]. All three SSL (6 cycle/min)-treated groups showed significant decreases in the time to cross the beam compared with the non-lighting group (*P* < 0.01 or 0.001). Animals in the 100 and the 200 Lm groups spent less time to cross the beam than those in the 50 Lm group (*P* < 0.001) which still showed an increase in the time to cross the beam compared with the Sta controls (*P* < 0.001). No difference was observed in balance performance between the 100 Lm and the 200 Lm groups.

### SSL (6 cycle/min) significantly reduced rot-induced Fos protein expression in the vestibular-autonomic nuclei

**Figure 6:**
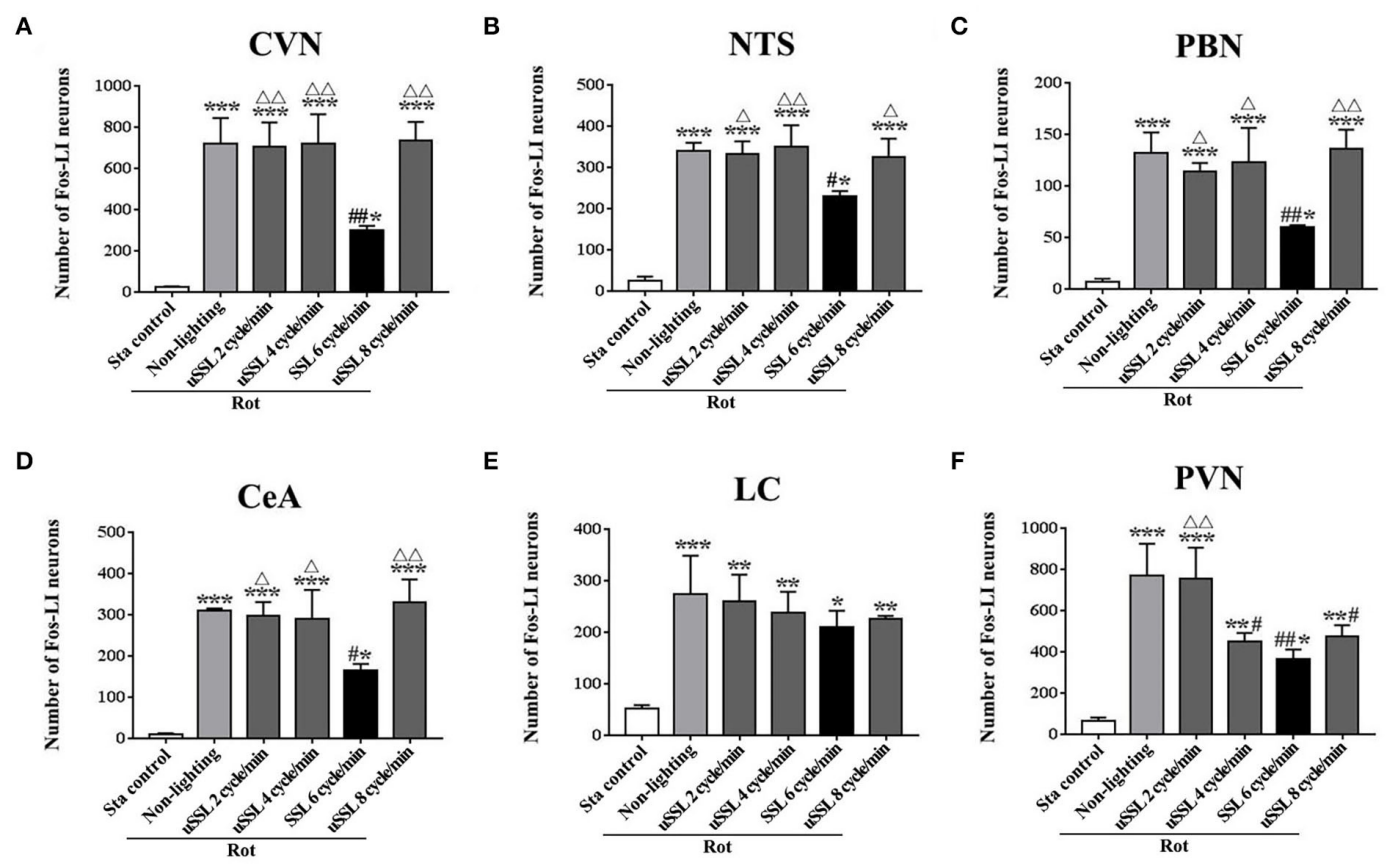
Number of Fos-labeled neurons in the vestibulo-autonomic and stress-related areas in rats receiving synchronized or unsynchronized stroboscopic lighting at 100 Lm during rotation. Fos-labeled neurons were quantified in the caudal vestibular nucleus [CVN, **(A)**], the nucleus of solitary tract [NTS, **(B)**], the parabrachial nucleus [PBN, **(C)**], the central amygdala [CeA, **(D)**], the locus ceruleus [LC, **(E)**], and the paraventricular hypothalamus nucleus [PVN, **(F)**]. Rot, rotation stimulation; Sta, static control treatment; SSL, synchronized stroboscopic light; uSSL, unsynchronized stroboscopic light; data are expressed as mean ± S.E. *P < 0.01, **P < 0.01, ***P < 0.001 vs. the Sta control group; ^#^*P* < 0.05, ^##^*P* < 0.01 vs. the Rot non-lighting group. ^Δ^*P* < 0.05, ^ΔΔ^*P* < 0.01 vs. the 6 cycle/min Rot group.

**Figure 7:**
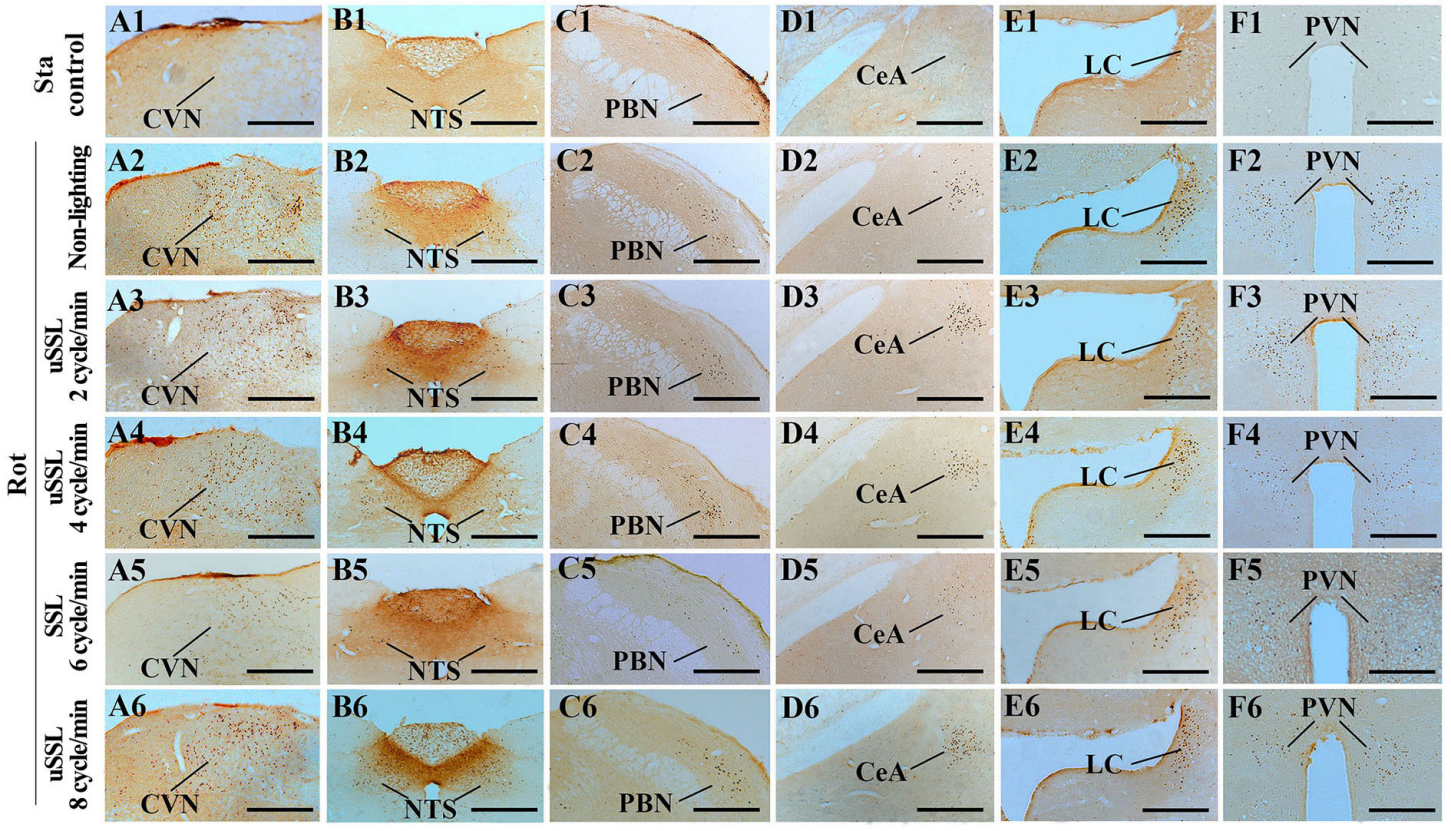
Representative photomicrographs showing Fos immunolabeling in the vestibulo-autonomic and stress-related areas in rats receiving synchronized or unsynchronized stroboscopic lighting at 100 Lm during rotation. Fos-labeling was presented in the caudal vestibular nucleus [CVN, **(A1–A6)**], the nucleus of solitary tract [NTS, **(B1–B6)**], the parabrachial nucleus [PBN, **(C1–C6)**], the central amygdala [CeA, **(D1–D6)**], the locus ceruleus [LC, **(E1–E6)**], and the paraventricular nucleus of hypothalamus [(PVN, **(F1–F6)**]. Rot, rotation stimulation; Sta, static control treatment; scale bars = 500 μm.

One-way ANOVA analysis revealed statistical differences among groups in the numbers of Fos-LI neurons in the CVN [*F*_(5,12)_ = 27.98, *P* < 0.001], the NTS [*F*_(5,12)_ = 46.71, *P* < 0.001], the PBN [*F*_(5,12)_ = 23.68, *P* < 0.001], the CeA [*F*_(5,12)_ = 29.59, *P* < 0.001], the LC [*F*_(5,12)_ = 10.50, *P* < 0.01], and the PVN [*F*_(5,12)_ = 22.69, *P* < 0.01]. *Post-hoc* analysis showed increased Fos expression levels in these nuclei in all Rot groups compared with the Sta control (*P* < 0.05, 0.01, or 0.001; Figures 6, 7). The numbers of Fos-LI neurons were significantly reduced in the CVN, the NTS, the PBN, and the CeA of animals receiving SSL (6 cycle/min) during rotation (Figures 6A–D, 7A5–D5) compared with the Rot non-lighting controls (*P* < 0.05 or 0.01; Figures 7A2–D2). Meanwhile, SSL-treated animals also had lower numbers of Fos-LI neurons (Figures 7A5–D5) than those treated with uSSL (*P* < 0.05 or 0.01; Figures 7A3–D3,A4–D4,A6–D6), but no difference was observed among the non-lighting Rot group and the uSSL groups. In contrast, all Rot groups, including the SSL group, the uSSL groups, and the non-lighting control, showed no significant difference in the numbers of Fos-LI neurons in the LC (Figures 6E, 7E2–E6). Nevertheless, animals treated with SSL and uSSL (4 and 8 cycle/min) had lower numbers of Fos-LI neurons in the PVN than the non-lighting controls (*P* < 0.05 or 0.01; Figures 6F, 7F2–F6), while animals treated with uSSL (2 cycle/min) had higher numbers of Fos-LI neurons than those treated with SSL (*P* < 0.01) but showed no difference when compared with the non-lighting controls.

## Discussion

This study showed differential effects of motion-synchronized and motion-unsynchronized stroboscopic lighting on whole-body Ferris wheel-like rotation-elicited gastrointestinal symptoms and motor disorders in rats. We found that SSL alleviated MS-related defecation and conditioned gaping as well as hypoactivity and balance disturbance, while uSSL was only effective for relieving defecation. The efficacy of SSL against MS behavioral responses was affected by peak light intensity but not color temperature. SSL but not uSSL suppressed rotation-induced neural activation in the vestibulo-autonomic nuclei (CVN, NTS, PBN, and CeA), while both SSL and uSSL decreased neural activity in the PVN. These results strongly suggested that MS induced by the conflict between vestibular and visual cues during passive motion could be alleviated by providing visual signals with the lighting phase that matches the motion pattern even without giving direct visual cues of the motion.

### Acceleration–deceleration Ferris wheel-like rotation in clockwise induced MS in rats

Acceleration (16°/s^2^)–deceleration(48°/s^2^) Ferris wheel-like rotation, known as the classical Crampton model, has been used for the production of MS in small animals for over three decades (Crampton and Lucot, 1985; Lucot and Crampton, 1991; Gallo et al., 1999; Xu et al., 2015, 2020). Sudden braking in a deceleration with the magnitude remarkably higher than acceleration was also effective in producing MS model for linear oscillation in humans (Vogel et al., 1982; Golding and Kerguelen, 1992; Butler and Griffin, 2006). Our previous studies showed that Ferris wheel-like rotation in a clockwise-counter-clockwise manner significantly induced MS-related responses including gastrointestinal symptoms (defecation and conditioned gaping) and motor disorders (hypoactivity and balance disturbance) in rats (Cai et al., 2010; Wang et al., 2012, 2015, 2017; Zhou et al., 2017; Manno et al., 2020). These behavior measurements have already been established to assess MS in rats or mice by other research groups (Ossenkopp and Frisken, 1982; Sutton et al., 1988; Ossenkopp et al., 1994; Yu et al., 2007; Limebeer et al., 2008; Bolognini et al., 2013; Tung et al., 2014; Zhang et al., 2021, 2022; Zhong et al., 2022) and were widely used as MS indices for drug evaluation in pharmacological experiments involving anti-MS agents such as anti-cholinergics (Wang et al., 2015; Qi et al., 2019), cannabidiolic acid (Bolognini et al., 2013), and Chinese traditional medicine (Zhang et al., 2021; Zhong et al., 2022). In this study, acceleration–deceleration Ferris wheel-like rotation in clockwise also triggered these MS-related behavioral responses with the provocative efficacy comparable to the well-established clockwise-counter-clockwise mode. Meanwhile, it successfully induced Fos protein expression in the rat CVN and autonomic area (NTS, PBN, and CeA), indicating enhanced neuronal activity in the vestibulo-autonomic pathways that is known to be essential for MS development (Pompeiano et al., 2002, 2004; Nakagawa et al., 2003; Ma et al., 2013; Balaban et al., 2014). This evidence suggested that the rotation mode and configuration utilized in this study can effectively establish MS model in the rats.

### SSL alleviated MS behavioral responses possibly via providing predictive visual cues of motion

Numerous evidence supports the idea that brain establishes an “internal model” of expected sensory information that represents normal motion pattern under 1 g gravity (Carriot et al., 2015). Predictive coding theory assumes that mismatch between “internal model” and real-time motor sensory inputs could produce prediction error on “what” happens in the brain under virtual or abnormal motion conditions and lead to sensory conflict (Arnal and Giraud, 2012; Kuiper et al., 2020; Nurnberger et al., 2021). On the contrary, expectancy of motion challenge was effective to enhance tolerance to MS in human subjects (Klosterhalfen et al., 2005, 2009) who showed definitely lower MS responses under directionally and temporally predictable motion conditions when compared with those under unpredictable ones (Kuiper et al., 2020). Moreover, when visual cue of motion was provided, MS could be alleviated due to the increased efficiency in motion anticipation that potentially reduced prediction error and visuo-vestibular conflict (Turner and Griffin, 1999; Bos et al., 2005; Krueger, 2011). For example, external forward view significantly reduced car sickness in car passengers with respect to no external forward view and blindfolded condition (Griffin and Newman, 2004). Subjects exposed to external viewing showed less sickness when receiving fore-aft and pitch oscillation motion than those exposed to either internal viewing or blindfolded (Butler and Griffin, 2009). A phase II clinical trial also showed that wearing an eyewear-mounted display providing a visual fixation target coupled with an artificial horizon that aligned with user movement was beneficial for MS-susceptible individuals in decreasing cardinal MS symptoms when riding public transport (Krueger, 2011). Similarly, artificial horizon visual information reflecting ship simulator movement also decreased MS symptoms during simulated sea voyage (Tal et al., 2012). In this study, we revealed that SSL (6 cycle/min) with intensity synchronized with angular velocity of rotation significantly decreased nausea-like conditioned gaping and motor disorders, although no real-time visual motion cue was provided. Our results are consistent with a previous finding that earth-fixed vision (stars in the sky) moving coherently with the simulator motion without providing real external view (e.g., rollercoaster track and earth horizon) significantly reduced MS severity compared with no vision condition (Feenstra et al., 2011). Our observation also echoes an early investigation showing that predictive visual cues produced by displaying moving point prior to visual stimulus enhanced self-motion perception and relieved MS in immersive virtual environment (Fujita, 2004). Webb et al. have reported that 8 Hz stroboscope illumination in cabin significantly reduced the incidence of nausea in helicopter passengers compared with non-stroboscope condition (Webb et al., 2013). The authors also claimed that the nausea scores were less under the 8 Hz setting than the 4 Hz setting which appears to reduce distorted vision-induced MS via transiently improving gaze stability (reduce retinal slip) during locomotion (Jones and Mandl, 1981; Bos and Bles, 2004; Han et al., 2005; Reschke et al., 2006; Webb et al., 2013; Muntaseer Mahfuz et al., 2018). However, as the nature of flight was not controlled, whether or not the vibration of helicopter matched the stroboscopic intensity remained unknown. Our study clearly showed that uSSL failed to relieve nausea-like conditioned gaping and motor disorders in rats. According to the multisensory integration theory, the weighting of visual cue might increase due to the unreliable of vestibular cue during passive motion stimulation (Angelaki et al., 2011; Carriot et al., 2015). As decreasing visual weighting relative to vestibular seems to be protective against virtual reality MS in physically stationary individuals when viewing unreliable and compelling visual scene, it is reasonable to speculate that increasing visual weighting to achieve visual-vestibular re-balance might help to counteract classical MS induced by unreliable vestibular cue of passive movement in the absence of corresponding visual input (Hettinger and Riccio, 1992; Weech et al., 2020). Based on this evidence, our results strongly suggested that providing stroboscopic light that provides visual cues relevant for motion pattern of the body might attenuate MS possibly via reducing brain prediction error when visual weighting is increased during passive motion stimulation. A limitation of this study is that only behavioral responses were observed for MS evaluation. As MS could affect thermoregulation leading to hypothermia in both humans and rodents (Nalivaiko et al., 2014), infrared imaging to detect changes in temperature of rats' tails should be utilized as objective assessment of MS in our future studies.

### SSL enhanced motor control ability and reduced rotation-induced postural instability

Greater postural sway in anteroposterior axis precedent to varieties of provocative environment exposure, such as ship motion, translational or rotational oscillations, and virtual reality, has been detected in subjects who later became motion sick compared with those who did not (Stoffregen et al., 2013, 2014; Koslucher et al., 2014, 2016). It indicated that unstable control of posture might be a precursor of MS and prolonged postural instability might play a role in induction of passive motion-induced MS (Riccio and Stoffregen, 1991). When experiencing MS, people often terminate working performance and try to keep immobile and stable by strapping the torso to the chair back or lying down on the back. Although body restraint might reduce postural instability, it could not alleviate MS sufficiently (Warwick-Evans et al., 1998). Nevertheless, there is evidence supporting the idea that augmented visual information could enhance active motor control (Rhea and Kuznetsov, 2017). Variations in visual demand could influence standing body sway (Stoffregen et al., 2000) and affect individual differences in MS incidence in humans (Stoffregen et al., 2013; Koslucher et al., 2016; Munafo et al., 2017). This study found that SSL (6 cycle/min) during rotation successfully reversed rotation-related motor disorders including hypoactivity and balance disturbance and simultaneously decreased MS nausea-like behavioral response in rats. Based on these results, we presume that postural stability might be disrupted by motion challenges, while motion-synchronized stroboscopic illumination may help to enhance motor control ability when visual-dependent prediction cues were more reliable than vestibular-dependent ones in provocative environments. However, as quadrupeds (e.g., rodents) seem to be more stable than bipeds (e.g., humans) in both anteroposterior and mediolateral body axes (Takahashi et al., 1991), whether postural instability could serve as a precursor of MS symptoms across species remains ambiguous. Given that continuous recording of postural control without interference during motion exposure could be achieved in humans (but not in rodents in this study), further investigations should be performed to observe the effect of SSL on postural instability that precedes the onset of MS symptoms to clarify the association between anti-MS efficacy and enhancement of motor control during motion challenge in humans.

### SSL suppressed neural activity in the vestibulo-autonomic nuclei during rotation

Previous studies have confirmed that the CVN neurons contribute to both cardiovascular control during head movements and autonomic manifestations of MS via its strong connections with autonomic areas, such as the NTS and the PBN in a variety of species (Balaban and Beryozkin, 1994; Yates et al., 1994; Balaban, 1996; Aleksandrov et al., 1998; Mori et al., 2005; Miller et al., 2008; Suzuki et al., 2012; Arshian et al., 2013; Gagliuso et al., 2019). Anatomical, optogenetic, and behavioral studies revealed that the PBN serves as a relay station for the CVN-PBN-NTS and the NTS-PBN-CeA pathways and mediates appetite suppression and gastrointestinal malaise in rodents (Balaban and Porter, 1998; Balaban, 2004; Carter et al., 2013, 2015; Alhadeff et al., 2015; Roman et al., 2016). Neural tract tracing studies have confirmed that the spinal vestibular nucleus of the CVN contains a large number of spinal cord-projecting neurons (some of which are inhibitory), forming vestibulospinal tracts that play important roles in maintaining postural stability (Valla et al., 2003; Liang et al., 2015). Our previous studies showed increased Fos protein expression in the CVN (especially the spinal vestibular nucleus), the PBN, the NTS, and the CeA of rats after receiving 2 h rotation (Cai et al., 2007, 2008) with great reproducibility and low individual variation and exhibited a large difference between rotation and static control group (5–10 fold change). Such stable and rigid responses of Fos protein expression have also been reported across species by other research groups, supporting using Fos protein as an indicator for vestibular activation in small sample size (*n* = 2–5) studies (Baizer et al., 2010; Balaban et al., 2014; Zhou et al., 2017; Samoudi et al., 2020). This study revealed that SSL but not uSSL reduced Fos expression induced by rotation stimulation in these nuclei. These results echo the behavioral observations showing that only SSL alleviated nausea-like gaping responses as well as hypoactivity and balance disturbance. Similar effects on MS-related Fos expression pattern in the CVN and the NTS have also been observed in both rats and Suncus murinus receiving anti-MS medications such as anti-cholinergics, anti-histamines, or ghrelin receptor agonists (Tu et al., 2017, 2020; Qi et al., 2019). These results suggested that stroboscopic light might decrease MS-related behavioral responses by inhibiting neural activity in CVN-connected autonomic pathways. The coordinated neural responses also echo the findings of an EEG study which showed the existence of MS response-correlated neural network signatures in human subjects based on the significant changes in global connectivity of the right-temporal-parietal, right-central areas, and right-frontal areas in MS-resistant individuals, but not in MS-susceptible ones, after the onset of vection (Wei et al., 2019). In addition, anatomical and electrophysiological studies found that the CVN neurons have poly-synaptic connections with the PVN which is a key regulator for autonomic, neuroendocrine, and behavioral responses to stress (Markia et al., 2008; Busnardo et al., 2013; Fadok et al., 2018). Surprisingly, we found that both SSL and uSSL significantly reduced Fos expression in neurons of the PVN but not the LC and simultaneously inhibited defection responses which might be the consequence of acute stress during initial period of rotation. Previous studies showed that stroboscopic lighting inhibited activation level in superior colliculus, thalamus, and auditory cortex of rat brain compared with ambient lighting (Soto-Montenegro et al., 2009), and the lighting with appropriate parameter could help to stabilize mood in humans and make people feel pleasant and relaxed (Park et al., 2013). This evidence suggested that stroboscopic light could possibly decrease neural activity in the PVN via inhibiting alternative brain areas besides the CVN and relieve general stress responses regardless of the source of stressors. The differential effects of SSL and uSSL on rotation-induced behavioral responses also supported the idea that general stress mediated by PVN activation seems to be a consequence of provocative motion rather than a causing factor of MS-specific autonomic responses such as nausea and vomiting (Otto et al., 2006).

## Limitations for practical application

Stroboscopic light (light flashes) has been demonstrated to be an external stimulus that can trigger photosensitive disorders (migraine and seizure) (Marmura, 2018; Okudan and zkara, 2018) and precipitate headaches (Martin, 2001, 2020); thus, the practical applicability seems to be limited especially in individuals sensitive to flickering lights. Nevertheless, it is well-known that low-frequency linear oscillation around 0.2 Hz frequency range is the prominent stimulus in provocating MS in a variety of modes of transport (land vehicles, ships, and aircraft) and the MS-related nausea responses (Golding and Markey, 1996; Golding et al., 2001). Application of low-frequency motion-synchronized stroboscopic light (<1 Hz), as was used in this study (6 cycle/min, 0.1 Hz), might not lead to serious photosensitive disorders which are most often seen when the frequency of flash stimulus is high (e.g., 15–25 Hz for induction of seizure) (Martin, 2001; Okudan and zkara, 2018). However, Duh et al. hypothesized that low-frequency visual motion oscillation at around 0.06 Hz was likely to provoke visually induced MS (Duh et al., 2004). Chen et al. also showed that visual oscillations along the fore-and-aft axis cause visually induced MS which appeared to be worse when increasing the frequency of visual oscillations from 0.0125 up to 0.8 Hz (Chen et al., 2016). We argue that stroboscopic light that provides no virtual motion cues could not produce vection which was the main trigger of visually induced MS during visual oscillation exposure. As flashes have been reported to distract observer's attention (Wright et al., 2015), the impact of stroboscopic light on working performance should be addressed especially for the application in personnel with important duty.

## Conclusion

Rotation-synchronized stroboscopic light alleviated MS-related nausea-like gaping behavior, hypoactivity, and balance disturbance as well as defecation responses in rats exposed to whole-body rotation in a light intensity-dependent manner. Such anti-MS effects might be related to the inhibition of neural activity in vestibulo-autonomic nuclei including the CVN, the NTS, the PBN, and the CeA. In contrast, unsynchronized stroboscopic light was only effective for reducing defecation response and neural activation in the PVN. These results suggested that stroboscopic light with emitting rate and intensity synchronized to motion pattern might possibly re-balance visuo-vestibular signals and enhance postural stability during passive motion stimulation. Our study strongly supports the utilization of motion-synchronous stroboscopic light as a potential countermeasure against MS-related autonomic symptoms such as nausea syndrome as well as locomotion and balance disorder in future.

## Data availability statement

The raw data supporting the conclusions of this article will be made available by the authors, without undue reservation.

## Ethics statement

The animal study was reviewed and approved by the Ethics Committee for Animal Experimentation of the Naval Medical University.

## Author contributions

YM, LP, and WL performed the behavioral tests. YM, LP, and SX completed the immunohistochemistry experiment. WL, LZ, and RQ conducted statistical analysis. YC, RQ, and JW were responsible for the design of the work. YC and YM were responsible for writing the manuscript. All authors read and approved the final manuscript.

## Funding

This work was funded by the Logistics Key project of PLA (BWS14J024) and the National Natural Science Foundation of China (81601638).

## Conflict of interest

The authors declare that the research was conducted in the absence of any commercial or financial relationships that could be construed as a potential conflict of interest.

## Publisher's note

All claims expressed in this article are solely those of the authors and do not necessarily represent those of their affiliated organizations, or those of the publisher, the editors and the reviewers. Any product that may be evaluated in this article, or claim that may be made by its manufacturer, is not guaranteed or endorsed by the publisher.
